# Reversible Cross-Linked Mixed Micelles for pH Triggered Swelling and Redox Triggered Degradation for Enhanced and Controlled Drug Release

**DOI:** 10.3390/pharmaceutics12030258

**Published:** 2020-03-12

**Authors:** Di Xiong, Liyang Wen, Shiyuan Peng, Jianchang Xu, Lijuan Zhang

**Affiliations:** 1 School of Chemical Engineering, Xiangtan University, Xiangtan 411105, China; 2 School of Chemistry and Chemical Engineering, South China University of Technology, Guangzhou 510640, China; cewenliyang@mail.scut.edu.cn (L.W.); cepengsy@mail.scut.edu.cn (S.P.); 201710103986@mail.scut.edu.cn (J.X.); 3 Postdoctoral Station of Chemical Engineering and Technology, School of Chemical Engineering, Xiangtan University, Xiangtan 411105, China

**Keywords:** mixed micelles, reversible cross-link, drug delivery, stimulus-responsive

## Abstract

Good stability and controlled drug release are important properties of polymeric micelles for drug delivery. A good candidate for drug delivery must have outstanding stability in a normal physiological environment, followed with low drug leakage and side effects. Moreover, the chemotherapeutic drug in the micellar core should also be quickly and “on-demand” released in the intracellular microenvironment at the tumor site, which is in favor of overcoming multidrug resistance (MDR) effects of tumor cells. In this work, a mixed micelle was prepared by the simple mix of two amphiphilic copolymers, namely PCL-SS-P(PEGMA-*co*-MAEBA) and PCL-SS-PDMAEMA, in aqueous solution. In the mixed micelle’s core–shell structure, PCL blocks were used as the hydrophobic core, while the micellar hydrophilic shell consisted of two blocks, namely P(PEGMA-*co*-MAEBA) and PDMAEMA. In the micellar shell, PEGMA provided hydrophilicity and stability, while MAEBA introduced the aldehyde sites for reversible crosslinking. Meanwhile, the PDMAEMA blocks were also introduced in the micellar shell for pH-responding protonation and swelling of the micelle. The disulfide bonds between the hydrophobic core and hydrophilic shell had redox sensitive properties. Reversible cross-linked micelles (RCLMs) were obtained by crosslinking the micellar shell with an imine structure. RCLMs showed good stability and excellent ability against extensive dilution by aqueous solution. In addition, the stability in different conditions with various pH values and glutathione (GSH) concentrations was studied. Then, the anticancer drug doxorubicin (DOX) was selected as the model drug to evaluate drug entrapment and release capacity of mixed micelles. The in vitro release profiles indicated that this RCLM had controlled drug release. In the simulated normal physiological environment (pH 7.4), the drug release of the RCLMs was restrained obviously, and the cumulative drug release content was only 25.7 during 72 h. When it came to acidic conditions (pH 5.0), de-crosslinking of the micelles occurred, as well as protonation of PDMAEMA blocks and micellar swelling at the same time, which enhanced the drug release to a large extent (81.4%, 72 h). Moreover, the drug release content was promoted further in the presence of the reductant GSH. In the condition of pH 5.0 with 10 mM GSH, disulfide bonds broke-up between the micelle core and shell, followed by shedding of the shell from the inner core. Then, the micellar disassembly (degradation) happened based on the de-crosslinking and swelling, and the drug release was as high as 95.3%. The MTT assay indicated that the CLSMs showed low cytotoxicity and good biocompatibility against the HepG2 cells. In contrast, the DOX-loaded CLSMs could efficiently restrain the proliferation of tumor cells, and the cell viability after 48 h incubation was just 13.2%, which was close to that of free DOX. This reversible cross-linked mixed micelle with pH/redox responsive behaviors is a potential nanocarrier for chemotherapy.

## 1. Introduction

Polymeric micelles are novel nanocarriers for drug delivery, whose nanoparticle sizes usually range from 10 to 200 nm [1,2,3,4,5]. Consisting of a core–shell structure, polymeric micelles have shown many advantages, such as increasing drug solubility efficiently, prolonging the circulation time in blood, keeping good stability and accumulating at tumor sites by means of the enhanced penetration and retention (EPR) effect [6,7,8]. For these advantages, polymeric micelles have drawn high attention in the biomedical field. However, there are still some shortages and bottlenecks lying in the way of drug delivery research, including poor stability during blood circulation as well as slow drug release at targeted tumor sites [9].

The formation of micelles is a thermodynamic process with an equilibrium between polymeric micelles and unimers; the critical point is known as the critical micellar concentration (CMC) value [10]. The micelles usually undergo extensive dilution during circulation in the bloodstream, resulting in a low polymer concentration far less than CMC and the subsequent disassembly of the micellar architecture [11]. Many recent studies have proved that reversible crosslinking of the micellar structure (core or shell) can enhance the stability of micelles against dilution efficiently [12,13,14,15]. In our early work, two reversible cross-linked micelles (RCLMs) were designed and synthesized based on the two copolymers, 4-AS-PCL-P(PEGMA-*co*-MAEBA) and PCL-SS-P(PEGMA-*co*-MABEA) [16,17]. The RCLMs showed excellent stability against 1000-fold dilution at pH 7.4. In contrast, fast de-crosslinking of micelles occurred in the simulated intracellular condition at the tumor site (pH 5.0 with 10 mM GSH). The de-crosslinking eliminated the possible inhibitory effect of the cross-linked structure on drug release. Even so, the drug release from micellar core needed to overcome the hydrophobic–hydrophobic interaction between the drug molecules and core-forming block, which was usually a very slow and uncontrollable process. In the earlier study, the authors found that the copolymeric micelles from 4-AS-PCL-P(PEGMA-*co*-MAEBA) had good stability but showed a limited drug release of just 71.5% in 108 h. Limited drug release means non-controlled drug release properties in targeted tumor sites, which might lead to more administration times or higher dosages for the potential application in pharmaceutics. Many studies have pointed out that the design of stimulus-responsive micelles based on the acid and reductive stimulus in the tumor microenvironment could overcome this bottleneck. The stimulus-responsive behavior usually includes stimulus-triggered swelling or disassembly (degradation) of the micellar architecture. For example, Yang and co-workers designed a pH-sensitive micellar system for targeted drug delivery based on the four-arm star copolymer 4-As-PCL-*b*-PDEAEMA-*b*-PPEGMA [18]. The PDEAEMA block containing tertiary amino groups in the side chain protonated when it encountered acid conditions in tumor cells (pH 5.0), followed by the transformation from hydrophobic blocks into hydrophilic ones. This hydrophilicity transformation led to the swelling or even disassembly of micelles, which was in favor of drug release with high speed. Shi and co-workers obtained amphiphilic micelles by self-assembly of the copolymer mPEG-SS-PzLL [19]. The micelle contained disulfide bonds between the micellar core and shell and could accurately respond to reductive conditions in tumor cells (10 mM GSH). The breaking up of disulfide bonds in the presence of 10 mM GSH resulted in the shedding of the micellar shell from the core, followed by the disassembly of micelles as well as enhanced drug release from the inner core. Their study indicated that this redox sensitive nanocarrier loaded with doxorubicin (DOX) could restrain the proliferation of tumor cells efficiently.

Aiming for efficient drug delivery and chemotherapy potential, the micellar carrier should have good stability against dilution as well as rapid intracellular drug release in tumor cells [20,21,22,23]. Among many methods for preparing multi-functional micelles, mixed micelle is a simple and effective method to obtain the micelles we need [24,25,26,27,28]. To obtain so-called mixed micelles, different copolymers are mixed in aqueous solution and self-assembled into micelles with various functions and advantages. Luo and co-workers prepared dual responsive mixed micelles by means of a simple mixture of the copolymers PCL-PDEA and mPEG-SS-PCL [29]. They found that the mixed micelles could respond to acid and reductive conditions with fast in vitro drug release and efficient antitumor effects. Many groups reported some other advantages of mixed micelles as well, such as enhanced drug entrapment, high targeting of the system, lower CMC values and so on [30,31,32,33].

**Scheme 1:**
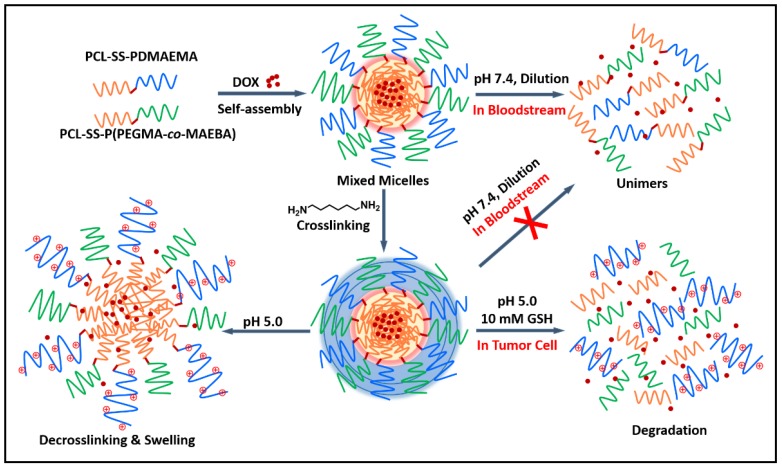
Schematic illustration for reversible cross-linked mixed micelles and the pH/redox-triggered controlled drug release.

In this work, we designed and prepared a novel mixed micelle by a combination of reversible crosslinking and pH/redox dual response to promote drug delivery efficiency. All these micellar structure designs and functional properties were based on the two micellar system of 4-AS-PCL-P(PEGMA-*co*-MAEBA) and PCL-SS-P(PEGMA-*co*-MABEA), aiming for good stability against extensive dilution along with highly controlled drug release behavior. The copolymers, PCL-SS-PDMAEMA and PCL-SS-P(PEGMA-*co*-MAEBA), were mixed in aqueous solution and self-assembled into mixed micelles. The micellar core consisted of a hydrophobic PCL block, which was approved by the FDA for biomedical use. In the micellar shell, PEGMA provided hydrophilicity and kept the stability of the micelle structure, and the aldehyde group containing an MAEBA unit introduced the reversible cross-linked site. The PDMAEMA blocks with tertiary amino groups in the shell could protonize in response to an acid pH. Moreover, disulfide bonds between the core and shell could rapidly break up in the presence of the reductant GSH, leading to disassembly of the mixed micelle. The reversible cross-linked mixed micelles were obtained by crosslinking the micellar shell with a cross-linker, namely hexamethylenediamine. Then, the particle size and stability of micelles were studied by the measurement of dynamic light scattering (DLS) and TEM. At the same time, DOX was used as the model drug to evaluate the drug entrapment capacity. An in vitro drug release experiment was carried out to study and analyze the release profiles in different simulated physiological environments. Finally, the cytotoxicity of drug-loaded micelles against HepG2 cells was also evaluated by MTT assay. Mixed micelles realized several functional properties with a simple mixed method for good stability by crosslinking, and a highly controlled drug release by means of dual stimulus-triggered micellar swelling and disassembly (Scheme 1). This reversible cross-linked mixed micelle was a potential drug carrier for tumor chemotherapeutics delivery.

## 2. Experimental Methods

The materials and measurement details are as follows: 2-(dimethylamino) ether methacrylate (DMAEMA, 99%, Aldrich, Shanghai, China) was passed through a column filled with basic alumina to remove inhibitors. Poly(ethylene glycol) methyl ether methacrylate (PEGMA, *M*_n_ = 500 Da, 99%, Aldrich) was purified by passage through a column filled with neutral alumina to remove inhibitor. ε-Caprolactone (ε-CL, 99%, Aldrich) was dried over calcium hydride and distilled under reduced pressure before use. Methacryloyl chloride (98%, Aldrich), dithiodiethanol (95%, Alfa Aesar, Tianjin, China), 1,1,4,7,10,10-hexamethyltriethylenetetramine (HMTETA, 99%, Aldrich), CuBr_2_ (99%, Aldrich), stannous octoate (Sn(Oct)_2_, 98%, Alfa Aesar), 2-bromoisobutyryl bromide (98%, Alfa Aesar) and pyrene (99%, Aldrich) were used. All other reagents were used as received. Then, the structures of the copolymers were investigated by Fourier transform infrared (FT–IR) spectrophotometer (Nicolet Nexus for Euro, New York, NY, USA). The spectra were taken from 400 cm^−1^ to 4000 cm^−1^. ^1^H NMR spectra of the copolymers and precursors were obtained by a Bruker AVANCE III 400 (Zurich, Switzerland) spectrometer operating at 400 MHz at room temperature. Number average molecular weight (*M*_n_) and polydispersity index (*M*_w_/*M*_n_) were measured by gel permeation chromatography (GPC) adopting an Agilent 1200 series GPC system (Agilent Technologies Inc., Santa Clara, CA, USA ) with THF as mobile phase with a flow rate of 1.0 mL/min at 30 °C. Morphologies of micelles were researched with transmission electron microscopy (TEM, Hitachi H-7650, Tokyo, Japan) operating at 80 kV. Confocal laser scanning microscopy (CLSM) images were obtained using a Leica SP8 microscope (Leica, Munich, Germany).

### 2.1. Synthesis of the Copolymers PCL-SS-P(PEGMA-co-MAEBA) and PCL-SS-PDMAEMA

The copolymer PCL-SS-P(PEGMA-*co*-MAEBA) and its precursor PCL-SS-iBuBr were synthesized according to our early report. PCL_27_-SS-iBuBr and PCL_27_-SS-P(PEGMA_10.2_-*co*-MAEBA_13.4_) in our early report [17] were selected and used in this work. The copolymer PCL-SS-PDMAEMA was synthesized according to the other report [34] based on the precursor PCL_27_-SS-iBuBr, which aimed to have the same hydrophobic block with PCL_27_-SS-P(PEGMA_10.2_-*co*-MAEBA_13.4_).

### 2.2. Critical Micellar Concentration (CMC) Measurement

The CMC values for the mixture of copolymer PCL-SS-PDMAEMA and PCL-SS-P(PEGMA-*co*-MAEBA) with different mass ratio were measured by the pyrene fluorescence probe technique. When the copolymeric concentration was up to the CMC value, copolymers could self-assembled into a core–shell micellar architecture. Then, pyrene could move and preferentially distribute in the hydrophobic micellar core, resulting in an obvious red shift on the third peak of the fluorescence excitation spectra. The copolymeric concentration corresponding to the red shift on the third peak was the micellar formation concentration, the so-called CMC. CMC values of the mixed copolymers with different mass ratio were measured and recorded, and the mass ratios of PCL-SS-PDMAEMA to PCL-SS-P(PEGMA-*co*-MAEBA) were 10/0, 7/3, 5/5, 3/7 and 0/10, respectively. In detail, a pyrene solution of (12 × 10^−5^ M) in acetone was evaporated, forming a thin film in the flask bottom. The aqueous solutions of copolymer mixture at various concentrations ranging from 0.0001 to 0.1 mg/mL were added to the pyrene-coated vials and stored in the dark overnight. Then, the above solutions were added to pyrene-filmed vials and stored at room temperature in the dark for 24 h. The CMC value was obtained based on the fluorescence excitation spectra of the mixed solution. During the measurement, the emission wavelength $\lambda_{em}$ was set to 373 nm to get the fluorescence excitation spectra ranging from 300 nm to 350 nm. Since the third peak of the excitation spectra shifted from 335.6 to 338.6 nm, the threshold concentrations where the ratios of $I_{338.6}$ to $I_{335.6}$ against copolymeric concentrations obviously increased could be thought as the CMC value.

### 2.3. Preparation of Mixed Micelles

The copolymeric mixed micelles were prepared by the dialysis method; the detail is as follows: For blank micelles, the mixture of PCL-SS-PDMAEMA and PCL-SS-P(PEGMA-*co*-MAEBA) (30 mg) was dissolved in DMSO (30 mL). Then the solution was transferred into a cellulose membrane bag (MWCO 3.5~4.0 KDa) and dialyzed against deionized water (pH 7.4) for 24 h. In addition, the preparation process of imine cross-linked micelles was conducted according to McCormick’s study [14]. The pH value of the blank micellar solution above was adjusted to 8.0–8.5 with the weak base NaHCO_3_. During the process, a pH meter was used to monitor the pH value change. Then, the small cross-linker hexamethylenediamine was added in the solution with stirring (amine/aldehyde mole ratio of 1/2). After stirring for 12 h, the resulting solution was dialyzed against deionized water (pH 7.4) for 24 h.

DOX-loaded cross-linked micelles were prepared by the same method as described in the blank micelle preparation with several steps. First, the model drug DOX·HCl was dissolved in DMSO (15 mL), and then 2-fold molar of TEA was added dropwise under stirring for 4 h in darkness to obtain free DOX. Subsequently, the mixture of PCL-SS-PDMAEMA and PCL-SS-P(PEGMA-*co*-MAEBA) (30 mg) was dissolved in DMSO (15 mL). Then, the two solutions were mixed and transferred into a cellulose membrane bag (MWCO 3.5~4.0 KDa) and dialyzed against deionized water for 24 h to remove the DOX, which was not entrapped in the micellar core. The pH value of the blank micellar solution above was adjusted to 8.0–8.5 with the weak base NaHCO_3_. Then, small cross-linker hexamethylenediamine was added in the solution with stirring (amine/aldehyde mole ratio of 1/2). After stirring for 12 h, the resulting solution was dialyzed against deionized water for 24 h to remove the unreacted crosslinker.

The deionized water for dialysis was replaced every 2 h in the first 12 h and every 6 h in the last 12 h. The obtained solution was filtered through 0.45 μm pore size filters, lyophilized and stored at −20 °C for further use. DOX-loaded micelles were dissolved in DMSO (10 mL), and the absorbance at 480 nm was recorded by using a UV–vis spectrophotometer (UV-2450, Shimadzu, Japan). Then, the concentration of DOX loaded into micelles was calculated according to the standard curve of DOX/DMSO solution. Drug loading capacity (LC) and entrapment efficiency (EE) were obtained according to the following equations:

(1) $$\mathrm{LC}\left(\%\right)=\frac{\mathrm{Weight}\;\mathrm{of}\;\mathrm{loaded}\;\mathrm{DOX}}{\mathrm{Weight}\;\mathrm{of}\;\mathrm{DOX}\;\mathrm{loaded}\;\mathrm{micelles}}\times 100\%$$

(2) $$\mathrm{EE}\left(\%\right)=\frac{\mathrm{Weight}\;\mathrm{of}\;\mathrm{loaded}\;\mathrm{drug}}{\mathrm{Weight}\;\mathrm{of}\;\mathrm{drug}\;\mathrm{in}\;\mathrm{feed}}\times 100\%$$

### 2.4. Stability Study of Mixed Micelles

The stability of mixed micelles was studied and evaluated by means of DLS measurement. In detail, the micellar solutions with a concentration 1 mg/mL (2 mL) was extensively diluted by deionized water (pH 7.4). The resulting solution was balanced for 24 h, and then the particle size and distribution were recorded by DLS measurement.

### 2.5. In Vitro Drug Release of DOX in Different Simulated Conditions

Controlled drug release at different conditions is a highlight of reversible cross-linked micelles. In this work, the DOX release profiles of the imine cross-linked micelles were studied in PBS solution with different pH values and GSH concentrations, i.e., (1) pH 7.4, (2) pH 5.0, (3) pH 5.0 with 10 mM GSH. The PBS solution with pH 7.4 was used to simulate the normal physiological environment (bloodstream), and the PBS with pH 5.0 in the presence of 10 mM GSH was used to simulate the intracellular condition in tumor cells. The DOX-loaded micellar solutions (3 mL) with a concentration of 1 mg/mL was added in dialysis bags (MWCO 3.5–4.0 KDa). Then these bags were immersed into the corresponding buffer solution (47 mL) with stirring at 37 °C ($V_{0}$ = 50 mL). After that, the solution outside the bag was collected (4 mL, $V_{e}$) for measurement of DOX concentration and replaced by the same volume with a fresh one. The DOX concentration was confirmed by UV–Vis spectrophotometer at 480 nm with a DOX standard curve.

Finally, the cumulative drug release percentage ($E_{r}$) was calculated from Equation (3).

(3) $$E_{r}\left(\%\right)=\frac{V_{e}\sum_{1}^{n}C_{i}+V_{0}C_{n}}{m_{DOX}}\times 100$$

where $m_{DOX}$ is the amount of DOX in micelles (mg), $V_{0}$ is the whole volume of the release medium (50 mL), $V_{e}$ is the volume of buffer solution collected from the dialysis bag ($V_{e}$ = 4 mL), $C_{i}$ represents the concentration of DOX in the $i^{th}$ sample and n is the replacing number.

### 2.6. MTT Assay

HepG2 cells (human hepatocellular carcinoma cells) were cultivated in DMEM with 10% FBS, streptomycin (100 μg) and penicillin (100 units/mL) at 37 °C in a humidified atmosphere containing 5% CO_2_. HepG2 cells were seeded in a 96-well plate with a density of 1 × 10^4^ cells/well and cultured with DMEM for 24 h. Blank micelles, DOX-loaded micelles and free DOX were dissolved in DMEM with a series of given concentrations. The fresh medium or pre-prepared sample solution was added in the plate and cultured for 24 h or 48 h. Next, 20 μL of MTT solution (5 mg/mL) was added into each well and subsequently incubated for 4 h. The medium was removed and replaced by DMSO, which was used to dissolve the internalized purple formazan crystals. A microplate reader was used to detect the solution absorbance at 570 nm. The cytotoxicity test was performed in replicates of six wells. The relative cell viability (%) was calculated with the following equation:

(4) $$\mathrm{Cell}\;\mathrm{viability}\;\left(\%\right)=\frac{A_{sample}-A_{blank}}{A_{control}-A_{blank}}\times 100\%$$

where $A_{blank}$ is the absorbance at 570 nm without cells, and $A_{control}$ and $A_{sample}$ are the absorbance at 570 nm in the absence and in the presence of sample treatment, respectively.

## 3. Results and Discussion

### 3.1. Synthesis and Characterization of Copolymers

**Figure 1:**
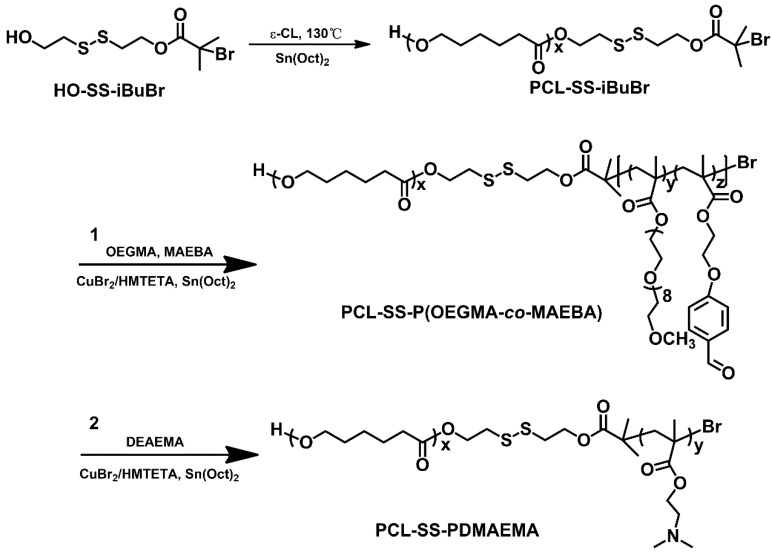
The synthetic route of the copolymer PCL-SS-PDMAEMA.

The precursor PCL-SS-iBuBr was selected as the macroinitiator. The synthesis route details of copolymers PCL-SS-P(PEGMA-*co*-MAEBA) and PCL-SS-PDMAEMA with different block ratios are shown in Figure 1.

**Figure 2:**
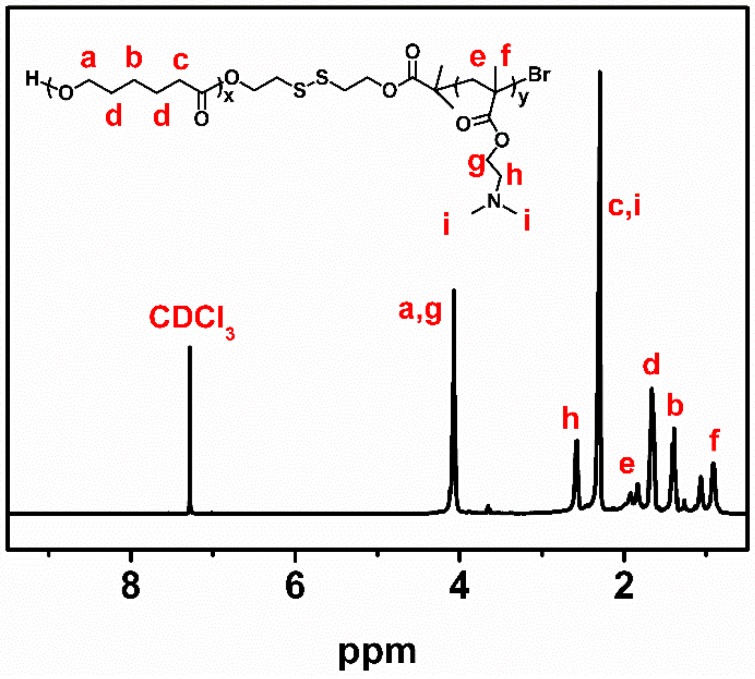
^1^H NMR spectra of PCL-SS-PDMAEMA.

The ^1^H NMR spectrum of PCL-SS-PDMAEMA is shown in Figure 2. The peaks appearing at 1.71–2.10 ppm (e) and 0.70–1.10 ppm (f) were assigned to the protons of the –C*H*_2_– and –C*H*_3_ on the copolymer backbone. The signal of –C*H*_2_– close to the tertiary amino group appeared at 2.54 ppm (h). The signal of –C*H*_3_ near the N atom (i) overlapped with the –CH2– signal (c) in the PCL backbone, which appeared at 2.26 ppm. The NMR spectrum indicated the successful synthesis of the copolymer. The degree of polymerization of DMAEMA was confirmed according to the integration ratio of the proton signal *h* to *b* as follows: $DP_{DMAEMA}=\frac{I_{h}}{I_{b}}\;\times \;{DP}_{PCL}$. Then, the number average molecular weight ($M_{n,NMR}$) of the copolymer PCL-SS-PDMAEMA could be calculated according to the following equation:

(5) $$M_{n,\;NMR}\;=\;6110\;+\;157\;\times \;\frac{I_{h}}{I_{b}}\;\times \;{DP}_{PCL}$$

where 157 is the molecule weight, $I_{b}$ and $I_{h}$ are the integral areas of peak *b* and *h*, respectively, and $DP_{PCL}$ is the degree of polymerization of ε-CL.

**Figure 3:**
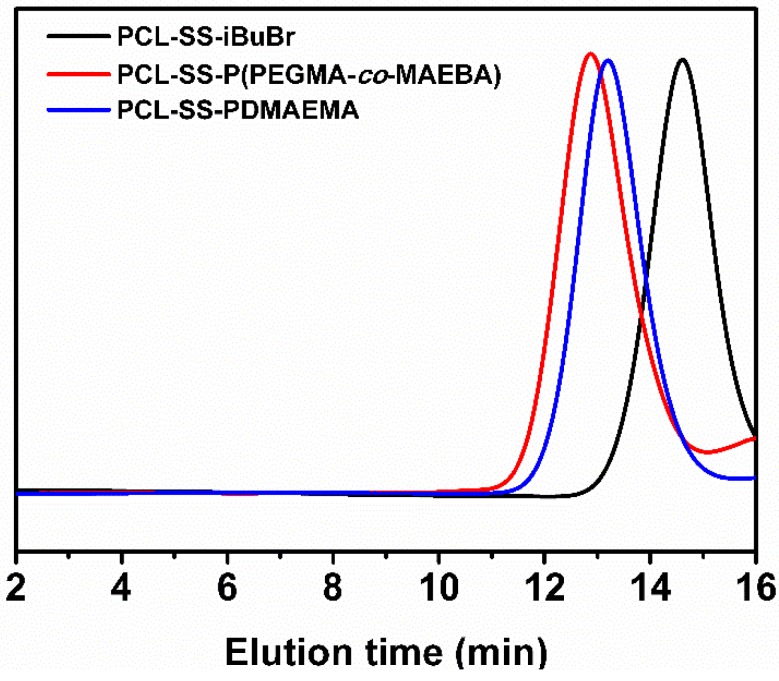
GPC (Gel Permeation Chromatography) traces of PCL-SS-P(OEGMA-*co*-MAEBA) and PCL-SS-PDMAEMA.

^1^H NMR and GPC data of the copolymers PCL-SS-PDMAEMA, PCL-SS-P(PEGMA-*co*-MAEBA) and their precursor PCL-SS-iBuBr are listed in Table 1. In addition, it is clear that GPC measurement results of the copolymers (Figure 3) were close to that of ^1^H NMR, indicating a relatively narrow distribution (*M*_w_/*M*_n_< 1.4) of the molecule weight and controlled copolymerization progress.

**Figure 4:**
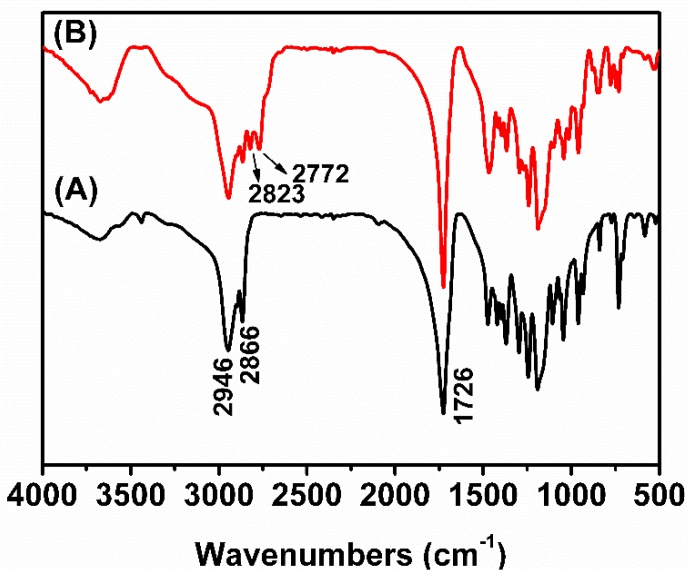
FT–IR spectra of PCL-SS-iBuBr (**A**) and PCL-SS-PDMAEMA (**B**).

The structure of the copolymers was further confirmed with FT-IR measurement. As shown in Figure 4, the absorption band of –CH_2_– in the copolymeric backbone appeared at 2867 cm^−1^ for stretching vibrations, and that of its bending vibrations appeared at 1465–1381 cm^−1^. The bands at 1726 and 1107 cm^−1^ were assigned to the stretching vibrations of C=O and C–O groups, respectively, in PCL-SS-PDMAEMA. The peaks at 2823 cm^−1^ (stretching vibrations) and 2772 cm^−1^ (bending vibrations) for C–H confirmed the presence of –N(CH_3_) group in the PDMAEMA block. In addition, there was no absorption at 1640 cm^–1^ in the FT-IR spectrum, indicating that the product was pure without methacrylate monomers.

### 3.2. Properties of Mixed Micelles

**Figure 5:**
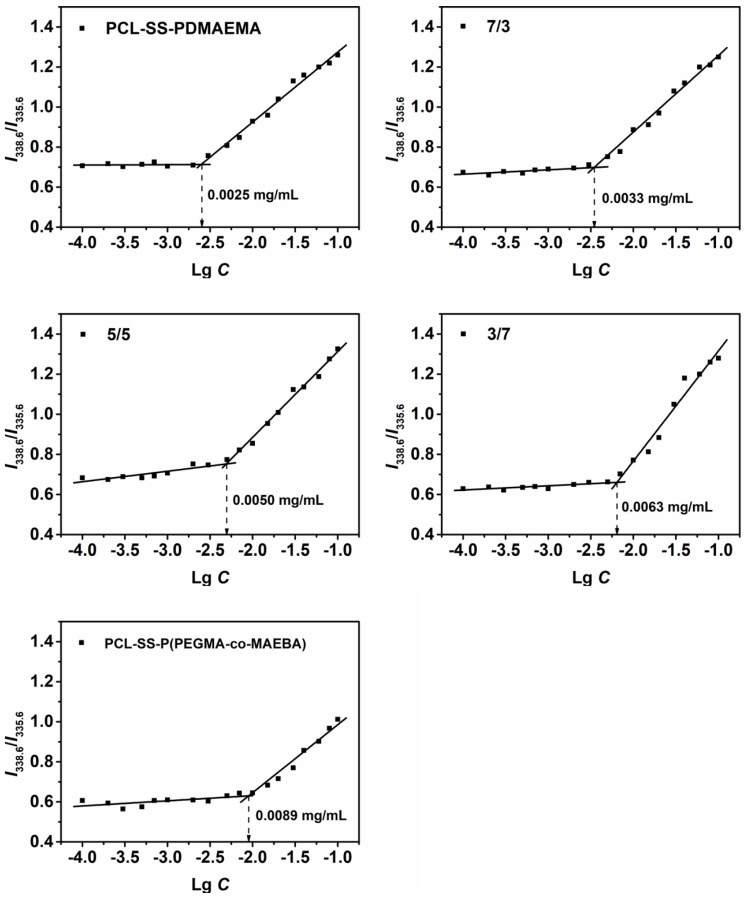
Graphs of intensity ratios (*I*_338.6_/*I*_335.6_) as functions of logarithm of copolymer mixture concentrations in aqueous solutions with different copolymeric mass ratios (PCL-SS-PDMAEMA to PCL-SS-P(PEGMA-*co*-MAEBA)) (The unit of C in the figures X-scale is mg/mL, and LgC is the corresponding logarithm of concentration C).

Critical micelle concentration (CMC) value is an important parameter of copolymer in diluted aqueous solution, indicating the stability of mixed micelles self-assembled from the copolymers. The CMC values with five different mass ratio of the copolymers PCL-SS-PDMAEMA and PCL-SS-P(PEGMA-*co*-MAEBA) were measured by the pyrene fluorescence probe technique. As shown in Figure 5, the copolymer PCL-SS-PDMAEMA had the lowest CMC value (0.0025 mg/mL), much lower than that of PCL-SS-P(PEGMA-*co*-MAEBA) (0.0089 mg/mL). When mixed with different ratios, the CMC values increased along with the rise of the mass ratios of PCL-SS-PDMAEMA to PCL-SS-P(PEGMA-*co*-MAEBA) (7/3, 5/5, 3/7), and were 0.0033, 0.0050 and 0.0063 mg/mL, respectively. Among the copolymer mixtures, the mixture with a ratio 7/3 had the lowest CMC value, indicating the highest stability in aqueous solution among these three mixed samples.

The hydrodynamic diameter distribution of mixed micelles self-assembled from the two copolymers with different ratios was confirmed by DLS measurement. As shown in Table 2, the smallest micellar particle size appeared at the copolymer ratio 7/3 and was 123.8 nm. When loaded with the model drug molecules, DOX, the particle size increased, which was on account of the hydrophobic DOX molecules entering into the micellar core by hydrophobic–hydrophobic interactions with the hydrophobic PCL blocks. Drug-loaded micelles had good size distribution (PDI < 0.350), proving that the drug loading had no obvious influence on the self-assembly progress. In addition, the mixed micelle at a 7/3 ratio had the highest drug loading capacity (LC, 16.4%) and entrapment efficiency (EE 58.9%). Since the mixed micelle at the 7/3 ratio had the lowest CMC value and highest drug loading capacity among the three different mixed micelles, it was used for further preparation and study of reversible cross-linked micelles.

**Figure 6:**
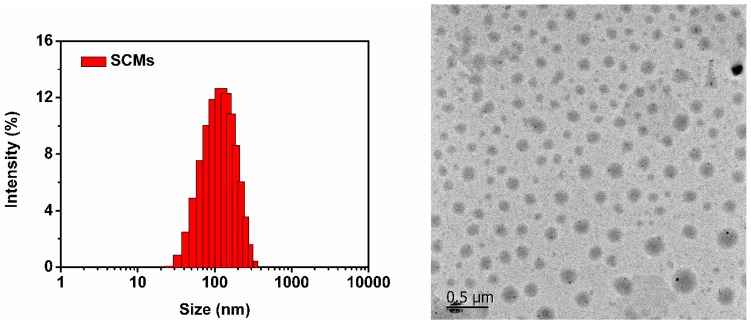
DLS results and TEM image of the cross-linked mixed micelles with DOX loading.

After crosslinking, the LC and EE of the cross-linked micelles were 15.8% and 56.3%, respectively, and some lower than that before crosslinking, which was due to the dialysis in crosslinking progress. The DLS result for drug-loaded micelles and the corresponding TEM image are shown in Figure 6. Obviously, the polymeric micelle distributed with sphere morphology, indicating a good control of the micellar crosslinking process.

**Figure 7:**
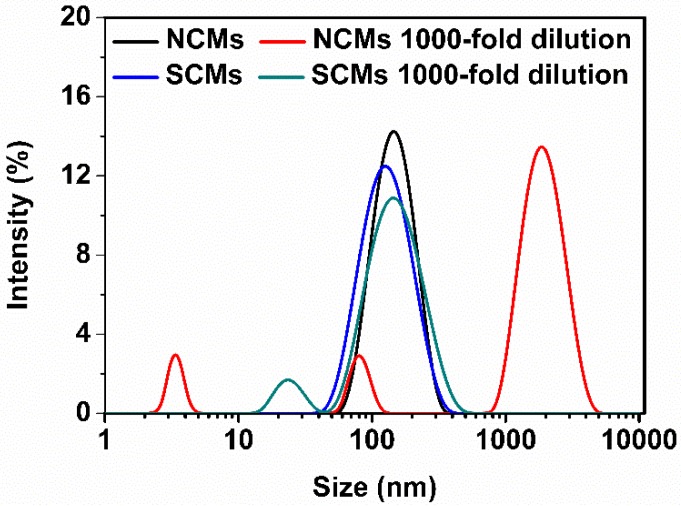
Size distribution of non-crosslinked micelles (NCMs) and reversible crosslinked micelles (SCMs) based on copolymer mixture before and after the 1000-fold water dilution.

The micellar stability against extensive dilution was studied by DLS measurement of the particle size change of the micelle solution with a concentration of 1 mg/mL. As shown in Figure 7, after 1000-fold dilution by water for 12 h, the particle size of the mixed micelles increased obviously and was up to 1000 nm, indicating a large disassembly and aggregation of the micelles. On the contrary, the cross-linked micelles just swelled a little with slightly broader size distribution and exhibited no significant change, proving that the reversible crosslinking of mixed micelle promoted the micellar stability against dilution efficiently.

### 3.3. DPD Simulation of Mixed Micelles

**Figure 8:**
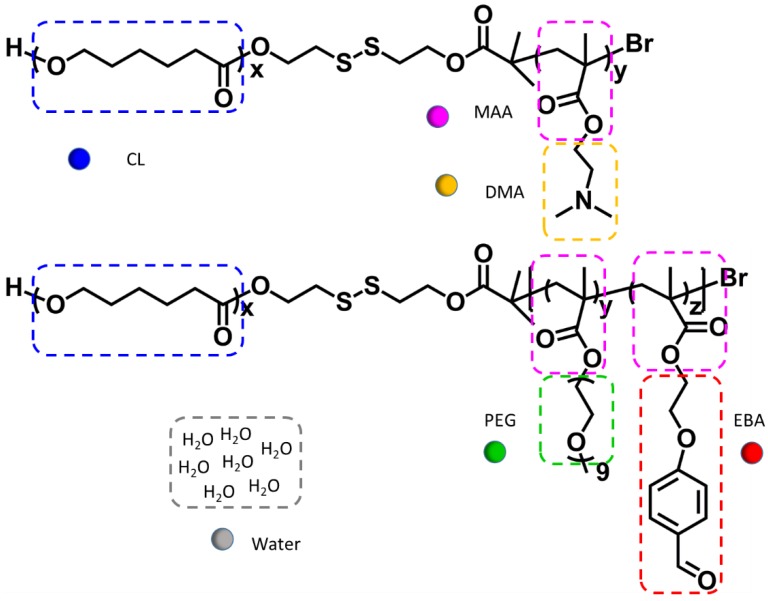
Molecular structures and coarse-grained models of the polymers PCL-SS-PDMAEMA and PCL-SS-P(PEGMA-*co*-MAEBA).

Dissipative particle dynamics (DPD) simulation has proven to be a useful tool for representing the self-assembly behavior of copolymers and micellar formation process [35,36]. In DPD, molecules are divided into sets of soft interacting beads and each bead represents a group of atoms that are macroscopically small but still large on the atomistic scale. For further study of the formation of the mixed micelles, the software tool Materials Studio 8.0 (Accelrys Inc., San Diego, CA, USA) was used and the DPD simulation method for block copolymers was introduced [32,37] to investigate the self-assembly of the copolymers and the mixed micellar morphology. In the simulation work, the two copolymers were divided into different types of beads, as listed in Figure 8, and the water bead responded to seven water molecules. The mass and radius of each bead were 112 amu and 3.7 Å on average, respectively, and 0.05 ns was set as the integration time step, and the total number of simulation steps was 100,000 steps.

As the coarse-graining was carried out above, the interaction parameters $a_{ij}$ between the different beads could be easily calculated and are shown in Table 3.

**Figure 9:**
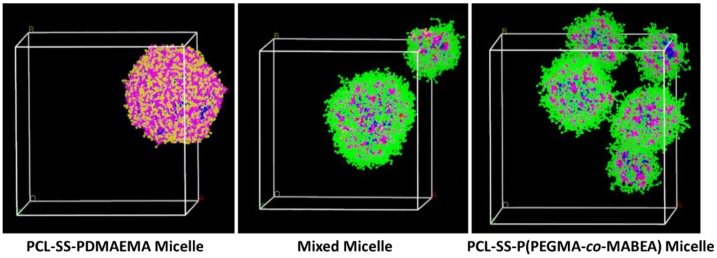
The micelles self-assembled from different copolymers and their mixtures, PCL-SS-PDMAEMA micelle, PCL-SS-P(PEGMA-*co*-MAEBA) micelles and the mixed micelles.

It was clear that the copolymers with one component, such as PCL-SS-PDMAEMA and PCL-SS-P(PEGMA-*co*-MAEBA), could formed well with sphere morphology (Figure 9). The micelles had core–shell architecture with PDMAEMA or PPEGMA as the hydrophilic shell and PCL as the inner core. When mixing the two copolymers, the mixed micelles could be also formed well with sphere morphology, which indicated that the copolymer mixture in aqueous solutions had little influence on the polymeric self-assembly process. The uniform sphere morphology might be in favor of the stability of mixed micelles and the following drug loading.

### 3.4. In Vitro Drug Release Profiles

**Figure 10:**
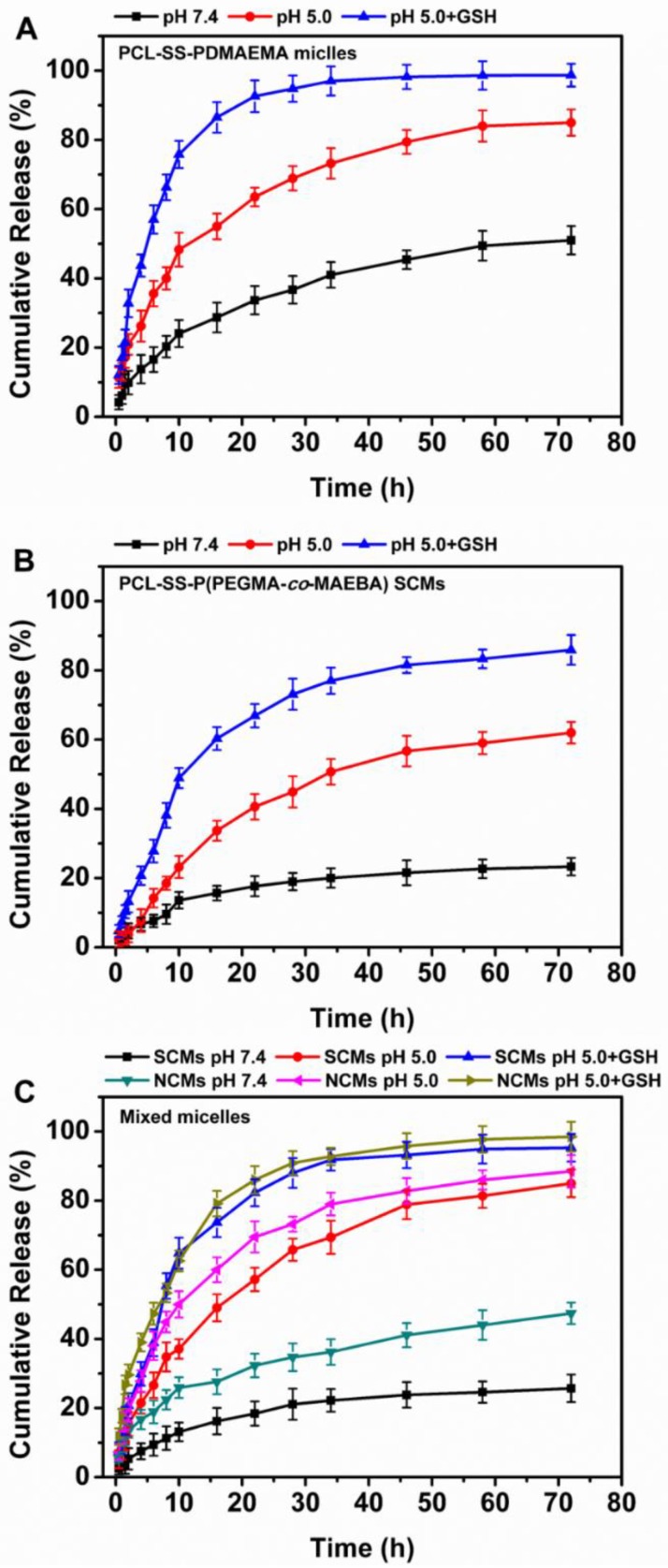
In vitro drug release profiles of PCL-SS-PDMAEMA micelles (**A**), PCL-SS-P(PEGMA-*co*-MAEBA) cross-linked micelles (**B**) and reversible cross-linked mixed micelles (**C**).

The in vitro drug release profiles of mixed micelles with or without crosslinking, and the micelles of the two copolymers were investigated and evaluated with different simulated conditions, such as bloodstream (pH 7.4), extracellular environment in tumor site (pH 6.5) and intracellular environment in tumor cells (pH 5.0 with 10 mM GSH). The concentration of the micellar medicament was 0.06 mg/mL, much lower than that of the DOX saturation concentration in PBS (0.51 ± 0.04 mg/mL at pH 7.4 and 1.04 ± 0.05 mg/mL at pH 5.0), which met the sink conditions, and there was no influence on the in vitro drug release results. As shown in Figure 10, in normal physiological environments such as in the bloodstream (pH 7.4), burst release appeared with PCL-SS-PDMAEMA micelles and the drug release amount was up to 51% (72 h). Analogously, the mixed micelles without crosslinking had a high leakage level with 47.4% as well. Contrary to the two micelles above without crosslinking, the drug leakage from the cross-linked mixed micelles was obviously restrained and declined to 25.7% within 72 h. The drug release gap at pH 7.4 indicated that the reversible cross-linked structure was an efficient barrier to prevent the release of drug molecules from the micellar core. When it comes to acidic conditions (pH 5.0), the drug release of cross-linked micelles of PCL-SS-P(PEGMA-*co*-MAEBA) increased obviously and was up to 62%, which was mainly for the breakage of the cross-linked structure with hydrolysis of imine. Drug release of PCL-SS-PDMAEMA micelles also rose up to 85%, and it was likely due to the fast protonation of the shell forming block PDMAEMA, followed by the hydrophilicity of the micellar shell and the swelling of micelles.

Moreover, the cross-linked mixed micelles had higher drug release amount at pH 5.0 (81.4%) based on the combination of micellar de-crosslinking and swelling. Different from the drug release at pH 5.0, the drug release of mixed micelles in the presence of 10 mM GSH was further promoted, and finally reached 95.3%, which was much higher than that of PCL-SS-P(PEGMA-*co*-MAEBA) cross-linked micelles and PCL-SS-PDMAEMA micelles. The big gap was mainly on account of the introduction of disulfide linkages between the hydrophobic and hydrophilic blocks, which could break-up with high concentration of GSH, leading to the disassembly of micelles. Thus, the stimulus-triggered de-crosslinking, swelling and disassembly of micelles in different conditions could be made use of to promote drug release in tumor cells efficiently.

### 3.5. MTT Assay

**Figure 11:**
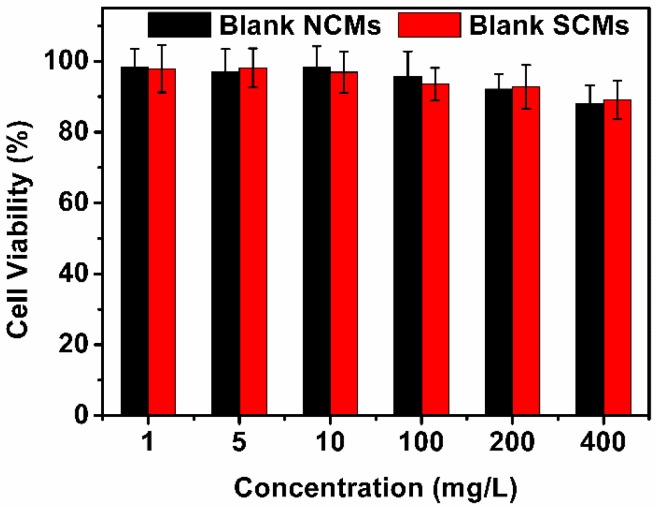
The in vitro cytotoxicity of blank micelles against HepG2 cells for 48 h.

**Figure 12:**
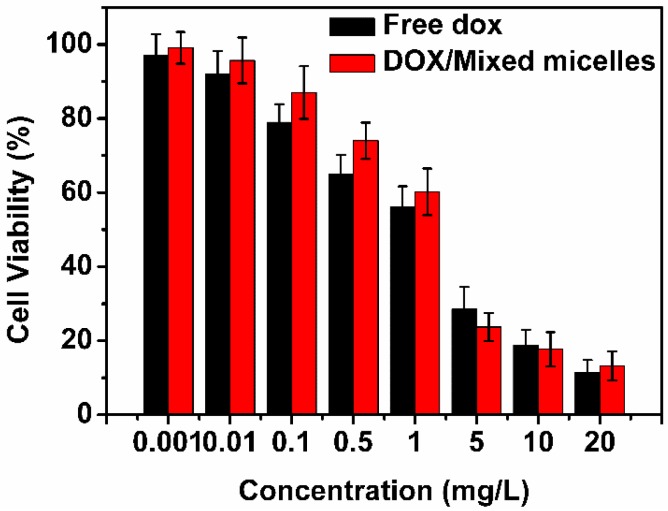
The in vitro cytotoxicity of free DOX and DOX-loaded mixed micelles against HepG2 cells for 48 h.

The materials for drug delivery should have low cytotoxicity, and this is the basic precondition. The cytotoxicity of the micelles in this work was evaluated with MTT assay, and the results are shown in Figure 11 and Figure 12. Obviously, the mixed micelles had much lower cytotoxicity before or after crosslinking, and the cell viability was higher than 85% even at the highest copolymer concentration with 400 mg/mL.

When treated with DOX-loaded mixed micelles and free DOX for 48 h, the cell viability was measured, as shown in Figure 11. The micelles and free DOX with low drug concentration had low cytotoxicity and relatively high cell viability. As the DOX concentration increased, both micelles and free DOX showed obvious cytotoxicity. The IC50 values for the DOX-loaded mixed micelles and free DOX were 2.27 mg/L and 1.95 mg/L for 48 h, respectively, and the cell viability decreased as the DOX concentration increased. At the concentration maximum with 20 mg/mL, the cell viability of DOX-loaded mixed micelles was only 13.2%, which showed a similar cytotoxicity as free DOX. The results indicated that the mixed micelles had low cytotoxicity, while showing high cytotoxicity against HepG2 cells after DOX loading.

## 4. Conclusions

In this work, the mixed micelles were obtained from the self-assembly of the mixture of copolymers PCL-SS-PDMAEMA and PCL-SS-P(PEGMA-*co*-MAEBA) in aqueous solution. The mixture with feed ratio of 7/3 had the lowest CMC value (3.3 mg/L), and the smallest micellar size (123.8 nm) as well as the highest LC (16.4%) and EE (58.9%). After reversible crosslinking of the micellar shell, the reversible cross-linked mixed micelles (RCLMs) could efficiently restrain drug leakage from the micellar core in a normal physiological environment (pH 7.4), and the drug release amount was as low as 25.7% during 72 h. The RCLMs had good stability against extensive dilution in bloodstream circulation. The imine cross-linked structure played a similar role to that of hydrazone bound in RCLMs based on PCL-SS-P(PEGMA-*co*-MAEBA). While in the simulated intracellular condition in tumor cells (pH 5.0 with 10 mM GSH), de-crosslinking occurred with imine hydrolysis at acidic environments, followed with the disappearance of the drug release barrier. At the same time, the shell forming block PDMAEMA protonized quickly, leading to micellar swelling, and the drug release increased, resulting in a similar drug release profile with PCL-SS-PDMAEMA micelles. Moreover, a high concentration of GSH broke the disulfide linkage between the hydrophilic shell and hydrophobic core, which promoted the drug release in tumor cells because of micellar disassembly (95.3%). The mixed micelles showed excellent stability against extensive dilution, as well as fast and maximal drug release in intracellular environments in tumor cells compared to the other micelles with a single copolymeric component. MTT assay showed that the copolymer micelles in this work had lower cytotoxicity, but high cytotoxicity against HepG2 cells after loading DOX molecules. These pH/redox dual responsive RCLMs with imine cross-linked structures have great potential as antitumor drug carriers for efficient drug delivery.

## Figures and Tables

**Table 1:** ^1^H NMR and GPC data of the copolymers PCL-SS-PDMAEMA, PCL-SS-P(PEGMA-*co*-MAEBA) and their precursor PCL-SS-iBuBr.

Sample	*M*_n, GPC_ *^a^*	*M*_w_/*M*_n_*^a^*	*M_n,NMR_*	ε-CL	*DP*		
					DMAEMA	PEGMA	MAEBA
PCL-SS-iBuBr	3400	1.29	3400	27	-	-	-
PCL-SS-PDMAEMA	8750	1.39	8270	27	31	-	-
PCL-SS-P(PEGMA-*co*-MAEBA)	10,900	1.37	11,600	27	-	10.2	13.4

**Table 2:** Critical micellar concentration (CMC) values of the copolymer mixture and the *D*_h_, PDI, LC and EE of the mixed micelles.

Samples	CMC (mg/L)	Blank Micelles		DOX-Loaded Micelles			
		D_h_/nm	PDI	D_h_/nm	PDI	LC/%	EE/%
PCL-SS-PDMAEMA	2.5	117.1	0.336	147.7	0.308	18.7	69.0
7:3	3.3	123.8	0.304	149.3	0.247	16.4	58.9
5:5	5.0	144.5	0.259	153.0	0.286	15.1	53.4
3:7	6.3	161.0	0.341	177.9	0.319	13.3	46.0
PCL-SS-P(PEGMA-*co*-MAEBA)	8.9	169.5	0.293	184.0	0.333	10.4	31.1

Note: D_h_, hydraulic radius; PDI, Particle distribution index; LC, loading capacity; EE, entrapment efficiency.

**Table 3:** The interaction parameters $a_{ij}$ between the different beads.

*a_ij_*	CL	DMA	MAA	PEG	EBA	Water
CL	25.00					
DMA	26.13	25.00				
MAA	27.01	30.45	25.00			
PEG	37.10	46.44	28.48	25.00		
EBA	25.22	32.88	25.18	28.33	25.00	
Water	53.20	38.22	34.60	26.10	141.62	25.00
